# Supplementary material for: Content-rich biological network constructed by mining PubMed abstracts
Source: BMC Bioinformatics. 2004 Oct 8;5:147. doi: 10.1186/1471-2105-5-147 (PMC528731; doi:10.1186/1471-2105-5-147)
Supplement: Additional File 2 — The original results of the above study (non-essential files are deleted to keep the file size under the limit set by BMC bioinformatics). [file 1471-2105-5-147-S2.bz2 › chilibotAdditionalFile2/dip50/549ID7910967E10902/html/CD4_IL16.html]

 


 **CD4** and **IL16** 
  
Found 142 abstracts in PubMed, retrieved 50.  
 

 What does Google say? 
 PDF only 
| .edu only 

---

**Interactive relationship** (e.g. stimulation, inhibition, etc)

**Inhibitory relationship**- interleukin IL 16  [ **IL16** ]  can inhibit the activity of  **CD4**  T lymphocytes.  Ref: 12869037 Clin Exp Immunol, 2003
- BACKGROUND IL 16  [ **IL16** ]  has been described as a natural soluble  **CD4**  ligand with immunosuppressive effects in vitro.  Ref: 12569988 Clin Exp Allergy, 2002
- In contrast, in cultures of murine epidermis alone no blocking by anti  **CD4**  became obvious and in  **CD4**  deficient mice Langerhans cell migration in response to interleukin 16  [ **IL16** ]  was maintained.  Ref: 11348450 J Invest Dermatol, 2001
- Theophylline 10 3 M and KF19514 10 6 M inhibited  **CD4**  eosinophil migratory responses induced by IL 16  [ **IL16** ] , but 10 6 M dexamethasone did not.  Ref: 11574756 Int Arch Allergy Immunol, 2001
- In whole skin explant cultures blocking of the interleukin 16  [ **IL16** ]  effect was also achieved with a monoclonal antibody against  **CD4** , the receptor for interleukin 16  [ **IL16** ] .  Ref: 11348450 J Invest Dermatol, 2001
- Agonists selective for H 2 dimaprit, H 3 4 R alpha methylhistamine, and H 4 clobenpropit were capable of inducing the release of bioactive IL 16  [ **IL16** ]  because CD8 cell supernatants induced  **CD4**  cell migration, which was abrogated by an anti IL 16  [ **IL16** ]  antibody.  Ref: 12235264 J Pharmacol Exp Ther, 2002
- The decrease of  **CD4**  T cells in MM may be mediated by activation of CD8 T cells derived cytokine IL 16  [ **IL16** ] .  Ref: 12191564 Leuk Res, 2002
- The resulting cells responded to IL 16  [ **IL16** ]  in an in vitro chemotaxis assay, and this biologic response could be blocked by anti IL 16  [ **IL16** ]  and anti  **CD4**  Abs as well as by a competitive peptide inhibitor corresponding to a sequence in the C terminal domain of IL 16  [ **IL16** ] .  Ref: 11937573 J Immunol, 2002
**Stimulatory relationship**- Therefore, IL 16  [ **IL16** ]  activation of eosinophils is  **CD4**  mediated to elicit the extracellular release of preformed RANTES and eotaxin, which then in an autocrine fashion act on plasma membrane CCR3 receptors to stimulate both enhanced LTC 4 production and the preferential release of IL 4, but not IL 12, from within eosinophils.  Ref: 11971026 J Immunol, 2002
- The grass pollen season further increased IL 16  [ **IL16** ]  expression and also increased the number of  **CD4**  cells in placebo treated, but not in BDP treated, allergic rhinitis patients.  Ref: 11551252 Allergy, 2001
- Recently, epidermal cells in lesional AD were reported to contain increased messenger m RNA levels of IL 16  [ **IL16** ] , a cytokine that induces chemotactic responses in  **CD4**  T cells, monocytes, and eosinophils.  Ref: 11941319 J Allergy Clin Immunol, 2002
- **CD4**  is a recognized IL 16R, and accordingly anti  **CD4**  Fab, soluble  **CD4** , and a  **CD4**  domain 4 based IL 16  [ **IL16** ]  blocking peptide inhibited the actions of IL 16  [ **IL16** ]  on eosinophils.  Ref: 11971026 J Immunol, 2002
- CONCLUSION Our results demonstrate that increased expression of IL 16  [ **IL16** ]  in T cells and mast cells in active Crohn s disease is associated with increased numbers of  **CD4**  lymphocytes.  Ref: 11709514 Gut, 2001
**Neutral relationship**- Interleukin IL 16  [ **IL16** ]  selectively recruits  **CD4**  cells.  Ref: 11551252 Allergy, 2001
- the synthesized IL 16  [ **IL16** ]  induced migration of  **CD4**  T cells.  Ref: 11243696 Cell Immunol, 2001
- IL 16  [ **IL16** ]  was expressed by  **CD4**  and CD8 positive T cells.  Ref: 11709514 Gut, 2001
- BACKGROUND Interleukin 16  [ **IL16** ]  IL 16  [ **IL16** ]  acts highly chemotactic on  **CD4**  bearing cells.  Ref: 11556864 Arch Otolaryngol Head Neck Surg, 2001
- Interleukin 16  [ **IL16** ]  IL 16  [ **IL16** ]  attracts  **CD4**  cells to sites of inflammation and plays a role in the interaction of dendritic cells, T cells and B cells.  Ref: 11566629 Eur Cytokine Netw< MedlineTA>Eur Cytokine Netw, 2001
- Interleukin IL 16  [ **IL16** ]  is associated with chemotactic activity for  **CD4**  cells.  Ref: 11502664 Chest, 2001
- IL 16  [ **IL16** ]  is a  **CD4**  specific ligand, and RANTES is a C C type chemokine that targets monocytes and lymphocytes.  Ref: 12810540 Endocrinology, 2003
- CONCLUSIONS Interleukin 16  [ **IL16** ]  was mainly expressed in a typical  **CD4**  like pattern in human tonsils.  Ref: 11556864 Arch Otolaryngol Head Neck Surg, 2001
- IL 16  [ **IL16** ]  is an important early chemotactic factor for  **CD4**  lymphocytes.  Ref: 11264709 Immunol Cell Biol, 2001
- Interaction between human interleukin 16  [ **IL16** ]  and  **CD4**  receptor of HIV 1.  Ref: 11360689 Acta Pharmacol Sin, 2000
- Theophylline and selective PDE IV inhibitor may prevent airway inflammation by downregulating  **CD4**  expression on eosinophils and inhibiting eosinophil migration through  **CD4**  and IL 16  [ **IL16** ]  interaction.  Ref: 11574756 Int Arch Allergy Immunol, 2001
- IL 16  [ **IL16** ]  is a ligand and chemotactic factor for  **CD4**  T cells.  Ref: 12528894 Cell Res, 2002
- In accordance with other data in the medical literature, we conclude that the most important function of IL 16  [ **IL16** ]  is the chemoattraction of  **CD4**  cells.  Ref: 12001788 Allergy Asthma Proc< MedlineTA>Allergy Asthma Proc, 2002
- **CD4**  serves as a coreceptor for HIV and IL 16  [ **IL16** ] .  Ref: 12213222 Exp Cell Res, 2002
- Our data strongly suggest that  **CD4**  lymphocytes constitute the major cellular source for IL 16  [ **IL16** ] .  Ref: 11556864 Arch Otolaryngol Head Neck Surg, 2001
- CONCLUSIONS IL 16  [ **IL16** ]  is involved in the asthmatic process by recruiting  **CD4**  T cells and thus plays an important role in  **CD4**  cell dependent eosinophilic inflammation in asthmatic airways.  Ref: 11775918 Zhonghua Jie He He Hu Xi Za Zhi, 1999
- Here we show that these effects of  **CD4**  ligation were not observed when anti  **CD4**  antibody was replaced with another  **CD4**  ligand, interleukin 16  [ **IL16** ] , or when the anti CD3 and anti  **CD4**  antibodies were spatially separated by immobilization on different beads.  Ref: 12848849 Immunol Cell Biol< MedlineTA>Immunol Cell Biol, 1999
- Interleukin 16  [ **IL16** ]  IL 16  [ **IL16** ]  has been described as a main cytokine involved in  **CD4**  cell recruitment during inflammation.  Ref: 12169178 Allergy, 2002
- It was also found that the percentages of  **CD4**  and EG2 cells were all positively correlated to IL 16  [ **IL16** ]  levels.  Ref: 11775918 Zhonghua Jie He He Hu Xi Za Zhi, 1999
- Circulating levels of interleukin 16  [ **IL16** ]  IL 16  [ **IL16** ]  were measured, in addition to  **CD4**  T cell counts, plasma HIV RNA levels, and the delayed type hypersensitivity DTH response.  Ref: 11527808 Clin Diagn Lab Immunol, 2001
- AIMS Interleukin 16  [ **IL16** ]  IL 16  [ **IL16** ]  has been characterised as a chemokine with selective chemoattraction for  **CD4**  inflammatory T cells.  Ref: 11709514 Gut, 2001
- IL 16  [ **IL16** ]  is a multi functional cytokine that uses  **CD4**  as a receptor to signal diverse biological activities by target cells including T lymphocytes, monocytes and eosinophils.  Ref: 11727516 Expert Opin Biol Ther, 2001
- Interleukin 16  [ **IL16** ]  IL 16  [ **IL16** ]  has been reported to bind a natural soluble ligand for the  **CD4**  molecule.  Ref: 11408767 Int Arch Allergy Immunol, 2001
- CONCLUSIONS These data suggest that TNF alpha induced  **CD4**  eosinophils may contribute to eosinophil migratory responses induced by IL 16  [ **IL16** ] .  Ref: 11574756 Int Arch Allergy Immunol, 2001
- Recently the influx of  **CD4**  lymphocytes has been related to the up regulation of IL 16  [ **IL16** ]  in AEDS skin lesions.  Ref: 12169178 Allergy, 2002
- Interleukin IL 16  [ **IL16** ]  has been reported as playing an important role in the accumulation of  **CD4**  cells.  Ref: 11574756 Int Arch Allergy Immunol, 2001
- Interleukin 16  [ **IL16** ]  supports the migration of Langerhans cells, partly in a  **CD4**  independent way.  Ref: 11348450 J Invest Dermatol, 2001
- The first of these, interleukin 16  [ **IL16** ] , is an immunomodulatory cytokine involved in the regulation of  **CD4**  T cells.  Ref: 11967309 J Virol, 2002
- Recently, IL 16  [ **IL16** ]  was also reported to be a potent chemotactic stimulus for  **CD4**  T lymphocytes and eosinophils in asthma and other pulmonary diseases.  Ref: 11509331 Am J Respir Cell Mol Biol, 2001
- Interleukin 16  [ **IL16** ]  IL 16  [ **IL16** ]  is a cytokine which is mainly produced by CD8 T cells and induces chemotaxis of  **CD4**  T cells and monocytes.  Ref: 11999883 Lupus, 2002
- IL 16  [ **IL16** ] , IL 8, IP 10 and RANTES are participated in the accumulation of  **CD4**  T cell.  Ref: 12233067 Nippon Rinsho, 2002
- IL 16  [ **IL16** ]  plays a role in trafficking of several immune cells and may be a major chemotactic signal for  **CD4**  cells.  Ref: 12001788 Allergy Asthma Proc< MedlineTA>Allergy Asthma Proc, 2002
- IL 16  [ **IL16** ]  expression in LCs in patients with AD correlated with the number of infiltrating  **CD4**  cells r =.72, P =.0017 and was completely downregulated parallel to the clinical response of AD lesions to topical treatment with FK506.  Ref: 11941319 J Allergy Clin Immunol, 2002
- Local expression of IL 16  [ **IL16** ]  seems to play a significant role in the initiation and persistence of the inflammatory process in Crohn s disease, presumably by IL 16  [ **IL16** ]  mediated recruitment of  **CD4**  cells, mostly lymphocytes, into the bowel wall.  Ref: 11709514 Gut, 2001

**Non-interactive relationship** (e.g. studied together, co-existance, homology, etc.)

- Co treatment of peripheral blood mononuclear cells PBMC with IL 16  [ **IL16** ]  plus IL 2 or IL 15 in vitro selectively expands the population of  **CD4**  T cells.  Ref: 11727516 Expert Opin Biol Ther, 2001
- Furthermore, Mo and AM were separately exposed to the three PM size fractions and supernatants assessed for the T helper  **CD4**  lymphocyte chemoattractant interleukin 16  [ **IL16** ]  IL 16  [ **IL16** ] .  Ref: 12746131 J Toxicol Environ Health A, 2003
- Stimulation of  **CD4**  T cells with IL 16  [ **IL16** ]  primes cells to respond to IL 2, by upregulating the expression of IL 2 receptor p75 CD25.  Ref: 11727516 Expert Opin Biol Ther, 2001
- AIMS To examine if IL 16  [ **IL16** ] , a chemoattractant for  **CD4**  cells, is detectable in airway secretions of ventilated newborns.  Ref: 11893439 Early Hum Dev, 2002
- IL 16  [ **IL16** ]  mediated ligation of  **CD4**  expressed on CD8 T cells was found to induce an intracellular signal that directs migration of these cells in vitro.  Ref: 11756173 Blood, 2002
- The pollen season induced change in IL 16  [ **IL16** ]  expression and in  **CD4**  cells was significantly more pronounced in placebo than in BDP treated patients.  Ref: 11551252 Allergy, 2001
- The increased number of  **CD4**  lymphocytes correlated positively with the increased number of IL 16  [ **IL16** ]  positive cells in Crohn s disease.  Ref: 11709514 Gut, 2001
- The modulation of immune cell costimulatory molecules, CD80, CD86, CD40, and HLA DR, and the release of the  **CD4**  T cell chemoattractant IL 16  [ **IL16** ]  were also investigated.  Ref: 12804531 Clin Immunol, 2003
- MIP 1beta induced G protein activation was further increased by simultaneous stimulation of  **CD4**  with its natural agonist, interleukin 16  [ **IL16** ] .  Ref: 12531905 J Biol Chem, 2003
- The amount of infiltrating  **CD4**  cells was determined by immunohistochemistry and correlated with the corresponding IL 16  [ **IL16** ]  cell number by step sections.  Ref: 11709514 Gut, 2001
- The ability of costimulated CD8 cells to respond to interleukin 16  [ **IL16** ]  IL 16  [ **IL16** ] , a ligand that binds  **CD4**  and induces cellular chemotaxis, was examined.  Ref: 11756173 Blood, 2002
- BACKGROUND It is currently unknown if interleukin IL 16  [ **IL16** ]  exists in the lungs of ventilated infants, and because the predominant cells in the airways of infants with CLD are  **CD4**  macrophages, we hypothesized that IL 16  [ **IL16** ]  plays a role as a pro inflammatory mediator in lung inflammation.  Ref: 11893439 Early Hum Dev, 2002
- Relationship between  **CD4**  CD8 T cell ratio and T cell activation in multiple myeloma reference to IL 16  [ **IL16** ] .  Ref: 12191564 Leuk Res, 2002
- They are suggested to be responsible for the observed influx of eosinophils IL 5, IL 16  [ **IL16** ] , and MIP 1 alpha and  **CD4**  T cells IL 16  [ **IL16** ]  and MIP 1 alpha into the challenged allergic mucosa.  Ref: 11424872 Allergy Asthma Proc< MedlineTA>Allergy Asthma Proc, 2002
- METHODS We evaluated the effect of natural allergen exposure during a grass pollen season on IL 16  [ **IL16** ]  expression and number of  **CD4**  cells in nasal mucosa.  Ref: 11551252 Allergy, 2001
- Here, we show that activation of monocyte derived LC like dendritic cells LLDC through engagement of FcepsilonRI induces the expression of IL 16  [ **IL16** ] , a chemoattractant factor for dendritic cells,  **CD4**  T cells, and eosinophils.  Ref: 11714796 J Immunol, 2001
- Double immunostaining identified  **CD4**  T cells as the major cellular source of IL 16  [ **IL16** ]  expression.  Ref: 11556864 Arch Otolaryngol Head Neck Surg, 2001
- We hypothesize that the double immunostained  **CD4**  ir and IL 16  [ **IL16** ]  ir cells represent activated T cells.  Ref: 11556864 Arch Otolaryngol Head Neck Surg, 2001
- RESULTS Prior to the pollen season, the expression of IL 16  [ **IL16** ] , but not the number of  **CD4**  cells, was significantly higher in patients with allergic rhinitis than in healthy control subjects.  Ref: 11551252 Allergy, 2001
- IL 16  [ **IL16** ]  promotes leukotriene C 4 and IL 4 release from human eosinophils via  **CD4**  and autocrine CCR3 chemokine mediated signaling.  Ref: 11971026 J Immunol, 2002
- ELISA was used to determine the concentrations of IL 16  [ **IL16** ]  in sputum, and  **CD4**  T cells and activated eosinophils EG2 cells in sputum were detected by immunostaining.  Ref: 11775918 Zhonghua Jie He He Hu Xi Za Zhi, 1999
- Microglial cells were visualized by immunolabeling of proteins which are known to be expressed either constitutively or facultatively, such as CD68, major histocompatibility complex class II MHC II, leukocyte common antigen LCA, leukocyte chemotactic factor LCF  [ **IL16** ] , macrophage inhibitory factor related protein MRP 8, MRP14,  **CD4**  and allograft inflammatory factor 1 AIF 1.  Ref: 11307625 Acta Neuropathol Berl , 2001
- R5 tropic HIV 1 glycoprotein 120, but not interleukin 16  [ **IL16** ] , the natural agonist, or X4 tropic glycoprotein 120, inhibited MIP 1beta binding to CCR5 in the presence of monomeric and dimeric soluble  **CD4** .  Ref: 12878220 Biochem Biophys Res Commun, 2003
- **CD4**  eosinophil migration in response to IL 16  [ **IL16** ]  was mostly, if not fully, chemokinetic and this migration was significantly inhibited by Fab of anti  **CD4**  monoclonal antibody.  Ref: 11574756 Int Arch Allergy Immunol, 2001
- As IL 16  [ **IL16** ]  has been shown to regulate  **CD4**  cell recruitment and activation, and is detected early following airway challenge of individuals with asthma, this two step process for IL 16  [ **IL16** ]  production by epithelial cells may represent a rapid response mechanism in the orchestration of allergic airway inflammation.  Ref: 12594062 Am J Respir Cell Mol Biol, 2003
- Although  **CD4**  is not G protein coupled, pertussis toxin inhibited IL 16  [ **IL16** ]  induced eosinophil activation.  Ref: 11971026 J Immunol, 2002
- We reported previously that fibroblasts from patients with GD express IL 16  [ **IL16** ] , a  **CD4**  specific chemoattractant, and RANTES, a C C chemokine, in response to GD specific IgG GD IgG.  Ref: 12794168 J Immunol, 2003
- IL 16  [ **IL16** ]  is a  **CD4**  specific chemoattractant and RANTES is a C C type chemokine.  Ref: 11777993 J Immunol, 2002
- METHODS We evaluated the effects of theophylline, KF19514 a selective phosphodiesterase PDE IV inhibitor and dexamethasone on  **CD4**  expression on eosinophils and eosinophil migration in response to IL 16  [ **IL16** ] , a natural soluble ligand of the  **CD4**  molecule.  Ref: 11574756 Int Arch Allergy Immunol, 2001
- AIM To study the interaction between human interleukin 16  [ **IL16** ]  IL 16  [ **IL16** ]  and the receptor  **CD4**  T lymphocyte differentiation antigen of human immunodeficiency virus type 1 HIV 1.  Ref: 11360689 Acta Pharmacol Sin, 2000
- Because IL 16  [ **IL16** ]  is a ligand for  **CD4**  and or an undefined  **CD4**  associated protein, the ability of this multifunctional cytokine to regulate the development of human MCs basophils from nongranulated progenitors residing in cord or peripheral blood was evaluated.  Ref: 11937573 J Immunol, 2002
- There was a significant correlation between the change in IL 16  [ **IL16** ]  expression and the number of  **CD4**  cells.  Ref: 11551252 Allergy, 2001
- In combination with IL 16  [ **IL16** ] , the beneficial effects of IL 2 may be augmented and specifically targeted to  **CD4**  T cells.  Ref: 11727516 Expert Opin Biol Ther, 2001
- We have reported that the bronchial epithelial cell line BEAS 2B can produce interleukin IL 16  [ **IL16** ] , a potent chemoattractant for  **CD4**  T cells.  Ref: 11243696 Cell Immunol, 2001
- Effect of seasonal allergen exposure on mucosal IL 16  [ **IL16** ]  and  **CD4**  cells in patients with allergic rhinitis.  Ref: 11551252 Allergy, 2001
